# Treatment Activity, User Satisfaction, and Experienced Usability of Internet-Based Cognitive Behavioral Therapy for Adults With Depression and Anxiety After a Myocardial Infarction: Mixed-Methods Study

**DOI:** 10.2196/jmir.9690

**Published:** 2018-03-16

**Authors:** Emma Wallin, Fredrika Norlund, Erik Martin Gustaf Olsson, Gunilla Burell, Claes Held, Tommy Carlsson

**Affiliations:** ^1^ Department of Psychology Uppsala University Uppsala Sweden; ^2^ Department of Women's and Children's Health Uppsala University Uppsala Sweden; ^3^ Department of Public Health and Caring Sciences Uppsala University Uppsala Sweden; ^4^ Uppsala Clinical Research Center Uppsala University Uppsala Sweden; ^5^ Department of Medical Science Uppsala University Uppsala Sweden

**Keywords:** mental health, internet, cognitive behavioral therapy, computer-assisted therapy, myocardial infarction, attrition, adherence

## Abstract

**Background:**

Knowledge about user experiences may lead to insights about how to improve treatment activity in Internet-based cognitive behavioral therapy (iCBT) to reduce symptoms of depression and anxiety among people with a somatic disease. There is a need for studies conducted alongside randomized trials, to explore treatment activity and user experiences related to such interventions, especially among people with older age who are recruited in routine care.

**Objective:**

The aim of the study was to explore treatment activity, user satisfaction, and usability experiences among patients allocated to treatment in the U-CARE Heart study, a randomized clinical trial of an iCBT intervention for treatment of depression and anxiety following a recent myocardial infarction.

**Methods:**

This was a mixed methods study where quantitative and qualitative approaches were used. Patients were recruited consecutively from 25 cardiac clinics in Sweden. The study included 117 patients allocated to 14 weeks of an iCBT intervention in the U-CARE Heart study. Quantitative data about treatment activity and therapist communication were collected through logged user patterns, which were analyzed with descriptive statistics. Qualitative data with regard to positive and negative experiences, and suggestions for improvements concerning the intervention, were collected through semistructured interviews with 21 patients in the treatment arm after follow-up. The interviews were analyzed with qualitative manifest content analysis.

**Results:**

Treatment activity was low with regard to number of completed modules (mean 0.76, SD 0.93, range 0-5) and completed assignments (mean 3.09, SD 4.05, range 0-29). Most of the participants initiated the introduction module (113/117, 96.6%), and about half (63/117, 53.9%) of all participants completed the introductory module, but only 18 (15.4%, 18/117) continued to work with any of the remaining 10 modules, and each of the remaining modules was completed by 7 or less of the participants. On average, patients sent less than 2 internal messages to their therapist during the intervention (mean 1.42, SD 2.56, range 0-16). Interviews revealed different preferences with regard to the internet-based portal, the content of the treatment program, and the therapist communication. Aspects related to the personal situation and required skills included unpleasant emotions evoked by the intervention, lack of time, and technical difficulties.

**Conclusions:**

Patients with a recent myocardial infarction and symptoms of depression and anxiety showed low treatment activity in this guided iCBT intervention with regard to completed modules, completed assignments, and internal messages sent to their therapist. The findings call attention to the need for researchers to carefully consider the preferences, personal situation, and technical skills of the end users during the development of these interventions. The study indicates several challenges that need to be addressed to improve treatment activity, user satisfaction, and usability in internet-based interventions in this population.

## Introduction

### Background

Symptoms of depression and anxiety are common following a myocardial infarction [1,2]. These symptoms predict a worse somatic prognosis [3,4], and treatment and rehabilitation adherence [5,6], as well as poor quality of life [7]. Mental ehealth services such as guided internet-based cognitive behavioral therapy (iCBT) may improve access to acceptable, effective [8], and cost-effective interventions to reduce symptoms of depression and anxiety [8,9]. iCBT has also been found to improve psychological and physical functioning, as well as disease-related impact in chronic somatic conditions [10]. The use of eHealth solutions has received a growing interest as a suitable method in societies with limited health care resources and increasing numbers of aging individuals living with cardiac diseases [11] **.** There is preliminary evidence that iCBT may reduce symptoms of depression and anxiety among adults with high cardiovascular risk [12] **.**

Typically, guided iCBT uses a written treatment material and internet-based synchronous or asynchronous communication with a therapist [13]. Compared with the traditional psychological care delivered face-to-face, internet-based interventions have several advantages such as reduced costs and increased user control and convenience [14]. Offering therapy that is more accessible with regard to place and time has the potential to make it easier for patients to fit a therapy into their daily life [15] and work according to their own preferred pace [16]. Moreover, internet-based interventions may be a way to reach people who feel embarrassed when talking to a care provider about their symptoms [14]. However, preliminary evidence suggests that people of higher age, which is associated with myocardial infarction [17], may experience more technical problems using internet-based interventions [18]. It has also been suggested that participants recruited in routine care may have less favorable views of internet-based interventions [19] and that those recruited through a consecutive clinical procedure are less motivated to engage in internet-based interventions compared with patients recruited through self-referral [20].

Treatment acceptability may be defined as the extent intended users perceive a given intervention as reasonable, justified, fair, and palatable [21]. Studies investigating iCBT have reported issues with indicators of treatment acceptability [22], including low expectations of its helpfulness and credibility [23,24], low take-up rates [25], high dropout rates [20,26], and poor adherence [27]. Treatment activity is an important aspect for internet-based interventions that aim to treat depression and anxiety disorders, as number of completed modules correlate with outcome [28]. There is an articulated need for qualitative studies conducted alongside quantitative trials, which investigate determinants of treatment acceptability [25]. Studies that explore experiences of taking part in internet-based interventions may lead to valuable insights on how to offer more effective treatments [29,30]. As little is known about the acceptability of iCBT interventions among patients with a recent myocardial infarction, there is a need for explorative studies to investigate treatment activity and experiences among such intended end users.

### Objectives

The overall aim of this study was to explore treatment activity, user satisfaction, and usability experiences among patients allocated to treatment in the U-CARE Heart study, a randomized clinical trial of an iCBT intervention for treatment of depression and anxiety following a recent myocardial infarction (unpublished data, 2018; [31]). The following 2 research questions were addressed:

1. What was the treatment activity with regard to completed modules, completed assignments, and therapist communication initiated by the participants?
2. What positive and negative experiences of the intervention, as well as suggestions for improvement, did the participants describe?

## Methods

### Study Design

This study was conducted alongside the U-CARE Heart study. The results from the randomized controlled trial (RCT) indicate no differences between the groups in symptoms of depression and anxiety after intervention [31]. This study is a descriptive mixed methods study, with quantitative and qualitative approaches. The regional ethics committee in Uppsala approved the study protocol (2011/217). The RCT was preregistered at ClinicalTrials.gov, Identifier: NCT01504191 December 2011.

### The Internet-Based Cognitive Behavioral Therapy Intervention

**Figure 1:**
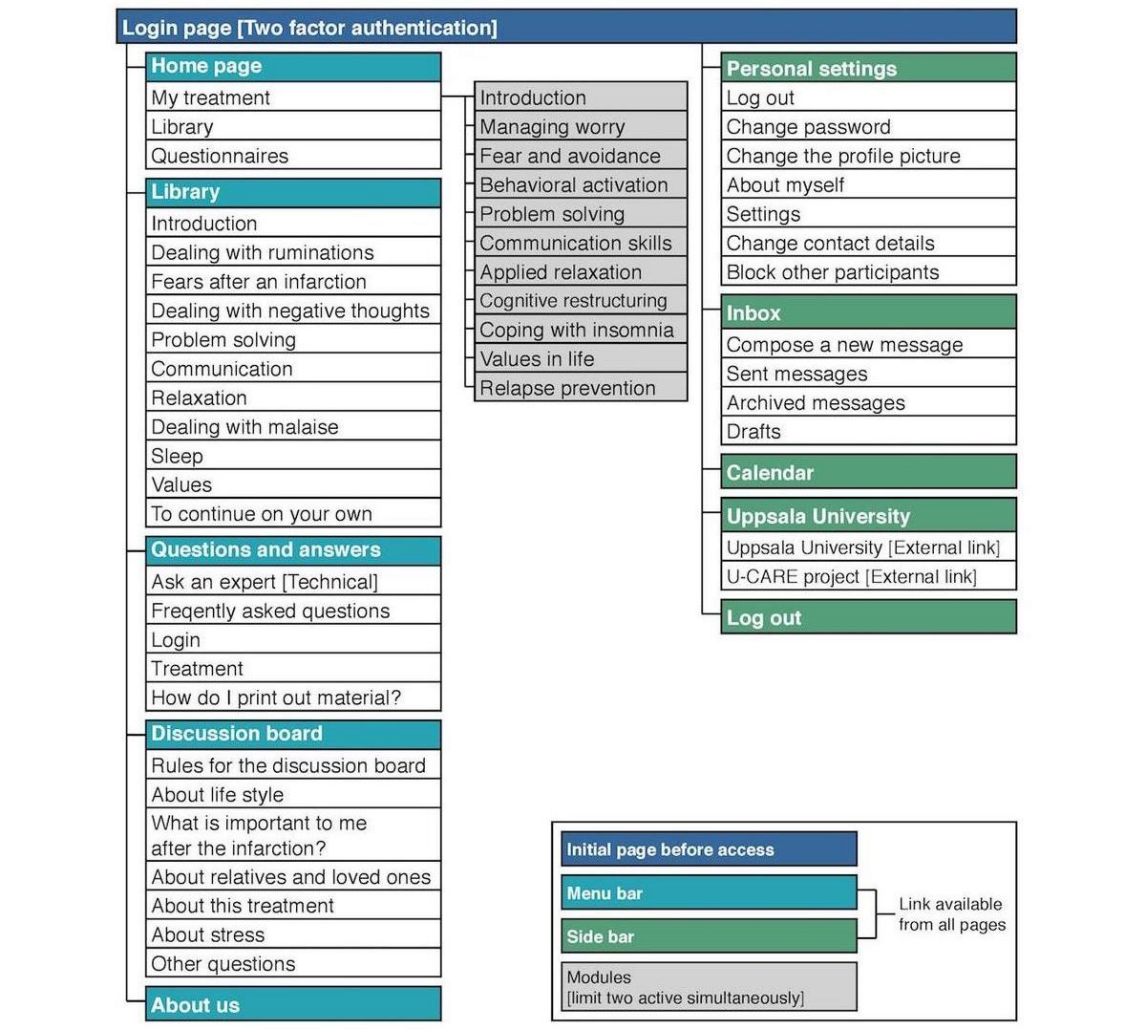
Sitemap of the internet-based portal.

The U-CARE Heart study used an internet-based portal to deliver an iCBT intervention tailored for patients with a recent myocardial infarction. A two-factor authentication solution with a password and numerical short message service (SMS) verification was required to log on to the portal. The design of the portal included a side bar and a menu bar, accessible from all pages. A short presentation and pictures of the therapist who worked in the program was provided in the “About us” section. Figure 1 presents a sitemap of the portal. Multimedia Appendix 1 presents a screenshot of the portal.

The treatment program consisted of 11 modules. Each module consisted of 2 to 4 steps. Each step contained 1 or 2 assignments, such as self-monitoring or registration of skills training. The treatment material consisted of PDF files with psychoeducation. The average word count per module was 6739.91 (SD 2786.79). Participants were encouraged to work with one step per week during the 14-week treatment period. The first introductory module was mandatory and oriented the user to the portal and the treatment program through an instructional video, psychoeducation about CBT, and common reactions post myocardial infarction. Thereafter, participants were invited to read a short description of the available modules, before choosing which modules to work with. Participants were limited to work with 2 active modules simultaneously. Supplementary material and video clips of interviews conducted with patients about their experience of depression and anxiety after a myocardial infarction were available throughout the course of treatment in an additional module called the Library. Participants also had access to a discussion board where they could communicate with other participants.

Each patient was assigned 1 of 3 therapists, who could be contacted any time. Therapists provided asynchronous written feedback on assignment via an internal message function within 24 hours. After completing all steps in a module, approval from a therapist was needed to activate a new module. Participants inactive for more than 1 week were reminded to stay active via phone calls. Participants unable to be reached were reminded by prompts sent via SMS.

Clinical psychologists and experts in IT solutions developed a preliminary version of the intervention. This version was evaluated through face-to-face think-aloud sessions [32], through consultations [33] with 6 test users with experience from emotional distress after a myocardial infarction, 2 stress management groups in regular cardiac rehabilitation, and two of the cardiac nurses involved in recruitment of patients at cardiac clinics. A description of the results of these consultations is presented in Multimedia Appendix 2. The introductory module was slightly modified (shortened) after a pilot trial [34]. During the study, the portal was adapted for handheld devices after 63 participants had been randomized.

### Recruitment

#### Randomized Controlled Trial

**Figure 2:**
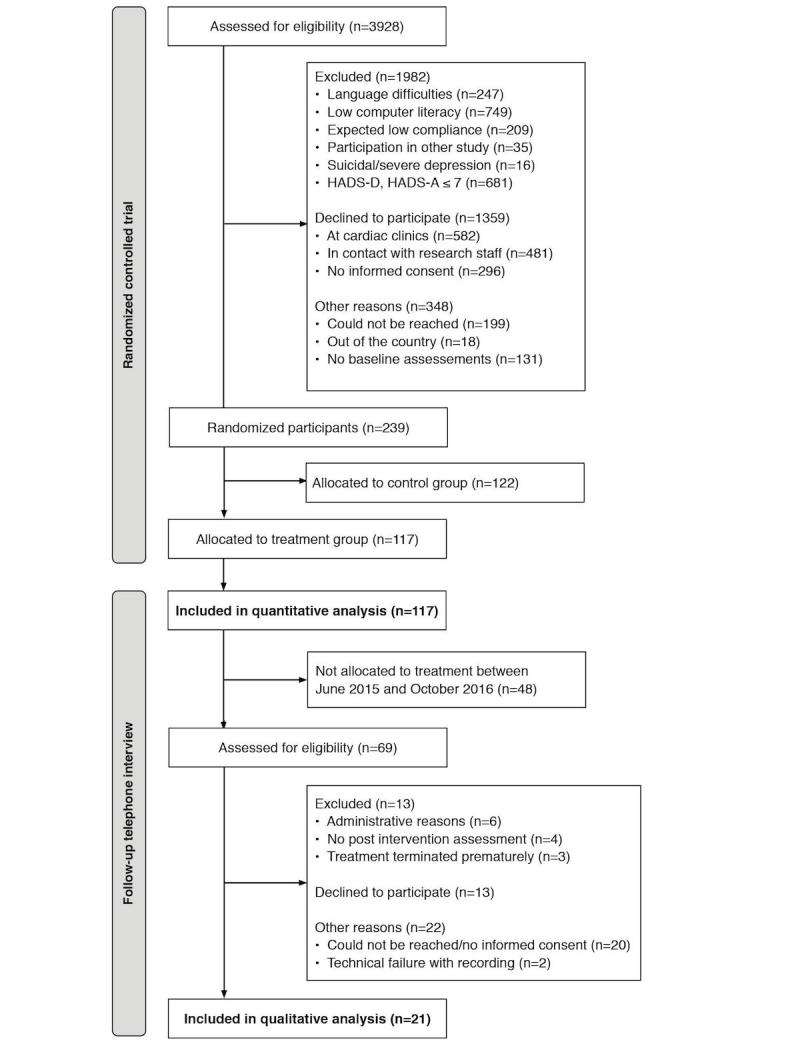
Recruitment of participants. HADS-A: Hospital Anxiety and Depression Scale-Anxiety; HADS-D: Hospital Anxiety and Depression Scale-Depression.

Patients were consecutively recruited from 25 cardiac clinics in Sweden. To be eligible, patients needed to: (1) be younger than 75 years, (2) have a medical history of a recent myocardial infarction less than 3 months prior, and (3) report a score >7 on either the depression or anxiety subscale in the Hospital Anxiety and Depression Scale (HADS) [35]. Potential participants were excluded if they: (1) had a life expectancy of less than 1 year, (2) were scheduled for bypass surgery, (3) were unable or unwilling to use a computer or mobile phone, (4) were unable to read or write in Swedish, (5) had an anticipated poor compliance to iCBT (eg, alcohol abuse), (6) had severe self-reported depression (total score >34) or risk of suicide (item 9 >3) on the Montgomery Asberg Depression Rating Scale Short form (MADRS-S) [36], or (7) participated in another ongoing trial with a behavioral intervention. The HADS and MADRS-S were administered via the internet. In total, 3928 persons were assessed for eligibility. Of these, 117 were allocated through randomization to the iCBT intervention (Figure 2).

#### Follow-Up Telephone Interviews

Participants (n=69) allocated to the treatment arm, between June 2015 and October 2016, were eligible to participate in a follow-up telephone interview (Figure 2). Participants were excluded if they had not filled out postintervention questionnaires in the RCT (n=4), or terminated treatment prematurely (n=3). Additionally, 6 participants were not approached because of administrative reasons. Of the approached participants (n=56), 13 declined participation, and 20 could not be reached or did not return the consent form. This resulted in 23 interviews. However, 2 interviews were excluded due to poor sound quality of the recordings, resulting in 21 interviews included in the qualitative analysis.

### Sample Characteristics

The majority of the participants in the randomized trial were males, employed, living in a relationship, born in Sweden, had no children in their household, and did not receive any current counseling (Table 1). The baseline sample characteristics for participants in the randomized trial were represented in the sample of participants included in the follow-up telephone interviews with 2 exceptions. None of the participants who were unemployed or on sick leave were included in the follow-up interviews. Furthermore, a higher proportion of the participants included in the interviews had studied at a university compared with participants not included in the interview (*P*=.03).

### Data Collection

#### Treatment Activity in the Randomized Controlled Trial

User activity was automatically registered through the U-CARE internet portal. Number of completed modules and assignments, and internal messages sent from patients to therapists, were used as quantitative measures of treatment activity.

#### Follow-Up Telephone Interviews

The fourth author (GB) conducted individual telephone interviews with the aid of a semistructured interview guide (Multimedia Appendix 3). Participants were informed that the purpose of the interviews was to evaluate their experiences of the intervention. Probes were asked to explore experiences and preferences. A preliminary interview guide was developed by three of the authors (EW, FN, and GB) and tested by interviewing 2 participants allocated to the intervention. These interviews were later included in the analysis. The phrasing of some of the questions in the guide was revised after these interviews. The interviews were audio-recorded, transcribed verbatim, and lasted between 22 and 66 min.

### Data Analysis

#### Treatment Activity in the Randomized Controlled Trial

Quantitative data regarding number of completed modules, assignments, and therapist communication initiated by participants were analyzed with descriptive statistics using R version 3.2.2 (R Foundation for Statistical Computing).

#### Telephone Interviews

The interviews were analyzed with inductive qualitative manifest content analysis, inspired by the outline presented by Graneheim and Lundman [37]. Interviews were transcribed verbatim by a professional transcribing agency. Two authors (EW and TC) were responsible for the analysis. Initially, the interview transcripts were read multiple times to obtain an overall perspective of the content. Meaning units were identified, defined as words, sentences, or paragraphs of a single message or context that corresponded to positive experiences, negative experiences, or suggestions for improvement. These meaning units were condensed, so that unnecessary words were removed. Thereafter, the condensed meaning units were labeled with a code that represented the core content and context of the meaning unit. Codes were sorted into categories and subcategories of the manifest content, defined as collections of codes that shared a commonality with regard to the visible content, identified with as little interpretation as possible. Initially, both authors worked independently with 2 interview transcripts and discussed the identified meaning units, condensed meaning units, codes, and preliminary categories. No impactful differences were observed. Thus, the first author (EW) identified meaning units, condensed the identified meaning units, and labeled these with a code for the remaining transcripts. Repeated face-to-face discussions were held between the authors EW and TC, with the purpose of scrutinizing the findings from the perspectives of the last author (TC), who had no previous experience of the U-CARE Heart intervention. Codes were sorted into subcategories and categories with the aid of NVivo version 11.3.2 (QRS International Pty Ltd., Australia). Multimedia Appendix 4 presents examples of the steps in the qualitative analysis, and Multimedia Appendix 5 presents backgrounds of researchers involved in qualitative data collection and analysis.

**Table 1:** Baseline demographic and clinical characteristics of participants. Between-group comparisons are conducted between participants not interviewed and interviewed. Categorical data is analyzed with Fisher exact test and continuous data is analyzed with Welsh *t* test.

Characteristics		Allocated to intervention (n=117)	Not interviewed (n=96)	Interviewed (n=21)	*P* value
**Demographic characteristics**					
	Age in years, mean (SD)	58.37 (8.98)	58.68 (8.67)	56.95 (10.38)	.48
**Sex, n (%)**					
	Female	44 (37.6)	37 (39)	7 (33)	.80
	Male	73 (62.4)	59 (62)	14 (68)	
**Occupation, n (%)**					
	Employed	78 (66.7)	62 (65)	16 (76)	.44^a^
	Unemployed	4 (3.4)	4 (4)	0 (0)	
	Retired	33 (28.2)	28 (29)	5 (24)	
	Sick leave	2 (1.7)	2 (2)	0 (0)	
**Highest educational level, n (%)**					
	Elementary	22 (18.8)	19 (20)	3 (14)	.03^b^
	High-school	45 (38.5)	41 (43)	4 (19)	
	University <3 years	24 (20.5)	18 (19)	6 (29)	
	University >3 years	26 (22.2)	18 (19)	8 (38)	
**Marital status, n (%)**					
	Single	18 (15.4)	15 (16)	3 (14)	>.99
	In relationship	99 (84.6)	81 (84)	18 (86)	
**Country of birth, n (%)**					
	Sweden	96 (82.1)	81 (84)	15 (71)	.21
	Other	21 (17.9)	15 (16)	6 (29)	
**Children in the household, n (%)**					
	Yes	43 (36.8)	33 (34)	10 (48)	.32
	No	74 (63.2)	63 (66)	11 (52)	
**Current counseling, n (%)**					
	Yes	30 (25.6)	24 (25)	6 (29)	.78
	No	87 (74.4)	72 (75)	15 (71)	
**Clinical characteristics, mean (SD)**					
	HADS-A^c^	10.27 (2.94)	10.39 (3.11)	9.76 (2.00)	.25
	HADS-D^d^	7.97 (3.15)	8.20 (3.26)	6.95 (2.42)	.05

^a^Employed versus other.

^b^Studied at university versus didn't study at university.

^c^HADS-A: Hospital Anxiety and Depression Scale-Anxiety.

^d^HADS-D: Hospital Anxiety and Depression Scale-Depression.

## Results

### Treatment Activity in the Randomized Controlled Trial

Of all participants allocated to intervention, 113 (96.6%, 113/117) initiated the introduction module, which was completed by 63 (53.9%, 63/117). Each of the remaining modules was completed by 7 or less of the participants. *Managing worry* and *Applied relaxation training* were the most frequently initiated and completed modules. The modules for *Communication training* and *Values in life* were not completed by any participant (Table 2).

**Table 2:** Number of participants in the randomized controlled trial (n=117) who initiated and completed the respective modules in the treatment program.

Module	Initiated, n (%)	Completed, n (%)
Introduction	113 (96.6)	63 (53.9)
Managing worry	23 (19.7)	7 (6.0)
Applied relaxation training	28 (24.0)	5 (4.3)
Behavioral activation	16 (13.7)	4 (3.4)
Fear and avoidance post myocardial infarction	7 (6.0)	3 (2.6)
Cognitive restructuring	11 (9.4)	2 (1.7)
Coping with insomnia	6 (5.1)	2(1.7)
Problem solving	4 (3.4)	2 (1.7)
Relapse prevention depression and anxiety	3 (2.6)	1 (0.9)
Communication skills	7 (6.0)	0 (0.0)
Values in life	3 (2.6)	0 (0.0)

**Table 3:** Total number of completed modules, completed assignments, and messages sent to therapist among the participants allocated to the intervention in the randomized controlled trial (n=117).

Number of completed modules, assignments, and sent internal messages at end of treatment period	Number of participants who completed modules, n (%)	Number of participants who completed assignments, n (%)	Number of participants who sent messages to therapist, n (%)
0	54 (46.2)	30 (25.6)	66 (56.4)
1	45 (38.5)	21 (17.9)	21 (17.9)
2	14 (12.0)	23 (20.5)	7 (6.0)
3	1 (0.9)	8 (6.8)	6 (5.1)
4	2 (1.7)	2 (3.4)	4 (3.4)
5	1 (0.9)	14 (12.0)	3 (2.6)
> 5	0 (0.0)	19 (16.2)	10 (8.5)

A minority of participants completed additional modules beyond the introductory module (18/117, 15.4%), completed more than 5 assignments (19/117, 16.2%), and sent more than 5 messages to the therapist (10/117, 8.5%; see Table 3).

**Figure 3:**
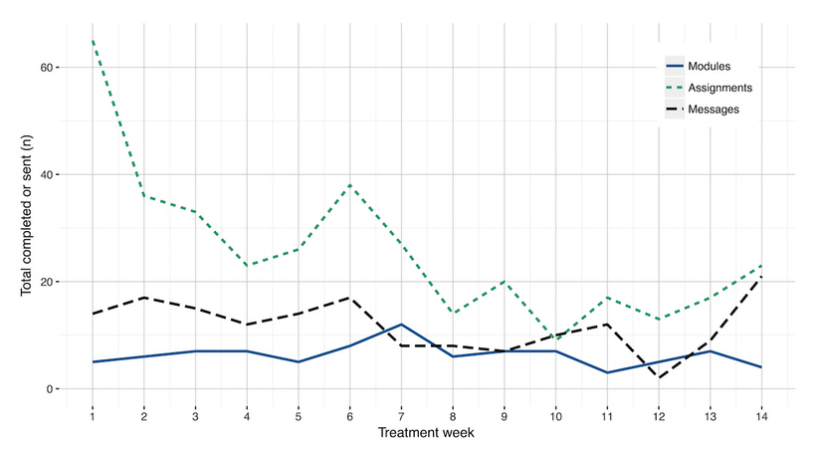
Total number of completed modules, assignments, and messages sent to therapist during the 14-week treatment period.

The mean number of completed modules, completed assignments, and messages sent to therapist did not reach above 0.6 at any of the 14 treatment weeks. The total summed range for all 14 weeks was 0 to 5 for completed modules, 0 to 29 completed assignments, and 0 to 16 messages sent to therapist (Multimedia Appendix 6). Most assignments were completed during the first week of treatment (Figure 3). Over the course of treatment, the total number of completed assignments and messages sent to therapist declined. A slight increase in treatment activity was observed in the middle of the treatment period, which coincided with the collection of outcome assessments. A slight increase in completed assignments and messages sent to therapist was observed toward the end of the treatment period. A total number of 41 out of 117 (35.1%) participants opened one or more supplementary material or a video clip in the library. Among these participants, the average number of opened items was 3.85 (SD 4.55).

### Telephone Interviews

We identified 4 main categories: (1) the portal, (2) the treatment program, (3) the therapist communication, and (4) the personal situation and required skills (Table 4). See Multimedia Appendix 7 for an expanded presentation of the qualitative results with the total number of participants who described experiences related to findings in respective category and illustrative quotes. In total, participants described 19 suggestions for improvement (Textbox 1).

**Table 4:** Summary of positive and negative experiences described in interviews.

Category and subcategory		Findings	
		Positive experiences	Negative experiences
**Portal**			
	Design	Appealing interface with easy navigation	Navigational difficulties, unfamiliar interface
	Usability	Easy and secure log-in procedure	Complicated log-in procedure with technical failures Required desktop or laptop, issues when using mobile device Cumbersome to open PDF files
**Treatment program**			
	Content of treatment material	Relevant, well-written, and useful information	Irrelevant outdated material and posts in discussion board Repetitive material with poor readability
	Working with the material	Manageable difficulty, approach gave time to reflect Time flexibility, possibility to select modules	Strenuous, tedious, difficult, and time-consuming work Too intensive work, restrictions in active modules felt rigid
	Treatment period	Deadline promoted activity toward end of treatment	Treatment duration and time to work with modules was too short
**Therapist communication**			
	Therapist feedback	Tailored, available, and rapid feedback Telephone conversations with therapist Reminders were useful prompt to log in	Lack of and irrelevant therapist feedback Aversive and stressful reminders
	Internet-based communication		Preference for verbal and synchronous communication Communication felt impersonal and involved a risk of misunderstanding
**Personal situation and required skills**			
	Unpleasant emotions evoked by the intervention		Bad conscience and guilt for being inactive Treatment rekindled difficult memories and emotions Fear of making mistakes
	Lack of time		Lack of time because of everyday life Poor timing of treatment
	Responding to outcome measures in questionnaires		Questionnaires were difficult to understand, felt repetitive, and irrelevant Strenuous work with questionnaires
	Technical aspects		Insufficient computer literacy Intervention required time in front of a computer Lack of Internet connection

**Summary of suggestions for improvement by category.**

Portal

- Remove the completed modules to facilitate navigation
- Include the possibility to have several windows open at the same time
- Make the portal available via CD-ROM and as an app for mobile devices

Treatment program

- Have less focus on depression and anxiety following a myocardial infarction
- Include the possibility to ask medical questions to health professionals and other participants in the portal
- Include information concerning how to communicate with children
- Use easy-to-read language
- Use closed-ended questions with predetermined alternatives in the treatment program
- Prolong the treatment period and allow longer time for work with modules that feel relevant for the patient
- Make the program feel more fun for the intended users

Therapist communication

- Offer synchronous verbal therapist communication, via telephone calls
- Offer more therapist feedback in decision making concerning which modules to work with
- Use audio or video recordings of therapist feedback
- Entitle the patient with their name instead of username
- Include picture of the therapist in all conversations

Personal situation and required skills

- Individualize the outcome questionnaires
- Make the outcome questionnaires easier to understand
- Allow participants to access previous responses in the outcome questionnaires
- Offer access to treatment closer in time to the infarction

## Discussion

### Principal Findings

Our study focused on treatment activity and user experiences of an iCBT intervention to reduce symptoms of depression and anxiety among adults with a recent myocardial infarction. The results show that treatment activity was low with regard to completed modules and assignments and submitted internal messages to therapists. Various positive experiences, negative experiences, and suggestions for improvements were described in follow-up interviews related to the internet-based portal, treatment program, therapist communication, as well as the personal situation and required skills of the participants. Previous research shows inconclusive and variable results concerning treatment activity and user satisfaction of iCBT. Although some studies report high levels of adherence and sufficient treatment satisfaction [8], others indicate that adherence varies considerably between studies [27]. Furthermore, it has been suggested that internet interventions may be more attractive among individuals who are familiar with computers, express confidence in writing about thoughts and feelings, who are attracted to the opportunity to reflect, and who appreciate the anonymity provided by the medium [15]. Our findings strengthen these assumptions and indicate a need for more research to investigate for whom, when, and how iCBT interventions may be a suitable treatment alternative to effectively alleviate symptoms of depression and anxiety after a myocardial infarction.

Although iCBT shows promise as a mode of treatment for symptoms of depression and anxiety [8], few studies have investigated these interventions through clinical consecutive recruitment [38]. This study included patients recruited in routine cardiac care, which provides new insights regarding treatment activity and user experiences of internet-based interventions. Our findings illustrate that although most participants initiated treatment, few persisted with the iCBT treatment. This finding indicates that the intervention was unable to successfully motivate the users to engage in the treatment. Compared with consecutive recruitment, self-referral recruitment strategies have the potential to identify individuals who persist with iCBT and who find the treatment effective [39]. Thus, it has been suggested that iCBT may only be acceptable among a subgroup of patients [20]. Another possible explanation of the observed low treatment activity may be that the participants did not feel a need for psychological treatment. For example, symptoms of depression and anxiety may be perceived as a normal reaction after a myocardial infarction. Patient attrition is an articulated issue for eHealth trials, which needs to be considered carefully when designing such interventions [26]. Our findings indicate a need for more research about how these patients experience a need for iCBT treatment, or if these types of interventions are better suited for certain subgroups of patients.

The observed low treatment activity and described negative experiences related to design and usability call attention to what has been described as a risk of distress and frustration when faced with technological difficulties [40]. In line with our findings, previous studies of iCBT interventions report that patients may experience struggles related to technology, delivery of treatment program, lack of support, and limited personalization of program content [41]. It is possible that our findings, in part, could be explained by the relatively high mean age among the participants. Higher age, as seen among patients with myocardial infarction [17], is associated with less use of the internet [42], low eHealth literacy [43], and unsuccessful skills needed to obtain reliable answers to health-related queries [44]. Moreover, older adults are more likely to report technological challenges in iCBT trials, and few studies have investigated user experiences of iCBT interventions for such populations [18]. The findings of this study illustrate the importance of efforts that aim to increase satisfaction and experienced usability among end users when developing eHealth interventions for patients with higher ages and a recent myocardial infarction.

Tailored interventions have the potential to successfully meet patient preferences by providing them with the choice of which treatment modules to work with [45] and adapting the treatment to the capacity of the patient [38]. In this study, the perceived positive and negative aspects varied considerably between individuals. This finding indicates a need to tailor interventions according to the intended end user’s individual preferences, personal situation, as well as computer skills. For example, participants who perceive text-based material strenuous and time-consuming to read may benefit from a less-extensive version of the intervention. Participants who find it difficult to write about thought and feelings may benefit from the use of closed-ended questions with predetermined alternatives in the treatment program. Telephone calls may be offered as an alternative to written feedback to participants with a preference for verbal and synchronous communication. One potential way to tailor the content according to individual needs of the intended users is to use patient and public engagement during the development phase [46,47]. In this study, patients with experience of emotional distress after a myocardial infarction and cardiac nurses were consulted about their views on the treatment material in the later stages of development. Consultations are considered to be lower levels of patient and public participation, as it may quickly lead to insights but lacks a commitment to subsequent actions [33]. It is possible that a different approach, involving collaborations with patients during the whole development process, could have led to an intervention closer in line with the preferences of the intended end users. In light of our findings, we acknowledge the potential importance of using high degrees of patient and public involvement when developing iCBT interventions.

### Limitations

In this study, there are methodological limitations that should be taken into consideration. The sample may not fully represent the population of patients with symptoms of depression and anxiety after a recent myocardial infarction. Patients were recruited in routine care at 25 Swedish cardiac clinics. Only patients below 75 years of age were invited to participate in the randomized trial. This may limit the generalizability and transferability with regard to older patients. The majority of the participants in the trial were males, employed, living in a relationship, and born in Sweden. Furthermore, only a subsample of those who took part in the intervention was interviewed. The reason for this was mainly practical, as we lacked necessary resources to collect qualitative data in the early stages of the study. This may imply a source of selection bias that may impact the results. We acknowledge that the qualitative results only reflect the experiences of a proportion of the whole sample in the RCT. Although the sample characteristics for participants in the RCT were represented in the sample of participants included in the follow-up telephone interviews, none of the participants who were unemployed or on sick leave were included in the follow-up interviews. Furthermore, a higher proportion of the interviewed participants had studied at a university compared with those who were not interviewed. This may imply a limited transferability to participants with lower levels of education. For example, it is possible that participants with experience of university studies may be more comfortable with text-based material and communication. Moreover, we did not collect any quantitative measure of computer literacy. Thus, we cannot make any claims about the actual computer literacy among the participants in our sample.

The data collection and analysis of the qualitative material may not fully represent the experiences of the interviewees. One psychologist who was not involved as a therapist in the treatment program conducted telephones interviews. Telephone interviews reduce the risk for socially desirable answers, may lead to increased sense of anonymity, and have the potential to make participants feel more comfortable [48,49]. On the other hand, telephone interviews make it impossible to observe nonverbal communication and create a comfortable physical setting where interviews take place [50]. We argue that the use of telephone communication and lack of previous contact with the interviewer promoted the participants to feel comfortable enough to be honest in their descriptions of their experiences and preferences. A semistructured interview guide with open-ended questions was used to cover our research questions, while still allowing for flexibility. The use of an interview guide implies instrumental consistency throughout the interviews [51,52]. Content analysis offers a systematic approach to describe patterns in text-based data [53,54]. However, there is always an embedded element of abstraction in qualitative analyses, which is impossible to completely disregard [51]. Thus, 2 authors with different backgrounds analyzed the data. We acknowledge that it is possible that potentially valuable information may have been lost due to potential biases or preconceptions.

### Suggestions for Future Research

The findings indicate a need for rigorous preparations before conducting iCBT interventions for adults with depression or anxiety after a recent myocardial infarction. There is a need for future research that investigates ways to ensure that development of these interventions is more adapted to the intended end users. The low treatment activity and negative experiences related to the use of the internet platform and the treatment content call attention to the importance of usability and feasibility trials. Future research should investigate patient, therapist, and treatment-related factors to improve treatment activity in internet-based interventions implemented in this population.

### Conclusions

Patients with symptoms of depression and anxiety after a recent myocardial infarction showed low treatment activity in guided iCBT with regard to completed modules, assignments, and messages sent to their therapist. They describe various negative experiences and suggestions for improvement, calling attention to the need for researchers to carefully consider the preferences, personal situation, and required skills of the end users during the development of these interventions. The findings indicate several challenges that need to be addressed to improve treatment activity, user satisfaction, and usability of internet interventions in this population.

## Appendix

Multimedia Appendix 1 Screenshot of the U-CARE Heart portal.

Multimedia Appendix 2 Description of development process.

Multimedia Appendix 3 Interview guide.

Multimedia Appendix 4 Examples of the steps in the qualitative analysis.

Multimedia Appendix 5 Backgrounds of the researchers involved in data collection and qualitative analysis.

Multimedia Appendix 6 Completed modules, completed assignments, and messages sent to therapist for each treatment week of the intervention.

Multimedia Appendix 7 An expanded presentation of the qualitative findings with illustrative quotes from interviews.

## Abbreviations

HADS: Hospital Anxiety and Depression Scale
HADS-A: Hospital Anxiety and Depression Scale-Anxiety
HADS-D: Hospital Anxiety and Depression Scale-Depression.
iCBT: Internet-based cognitive behavioral therapy
MADRS-S: Montgomery Asberg Depression Rating Scale Short form
RCT: randomized controlled trial
SMS: short message service
