# Supplementary material for: Treatment Activity, User Satisfaction, and Experienced Usability of Internet-Based Cognitive Behavioral Therapy for Adults With Depression and Anxiety After a Myocardial Infarction: Mixed-Methods Study
Source: J Med Internet Res. 2018 Mar 16;20(3):e87. doi: 10.2196/jmir.9690 (PMC5878371; doi:10.2196/jmir.9690)
Supplement: Multimedia Appendix 2 [file jmir_v20i3e87_app2.pdf]

A preliminary version of the interventions was evaluated by 6 test users with experience from emotional distress after a myocardial infarction, in one to three face-to-face think-aloud sessions through consultations. The test users provided feedback concerning the text, the clinical examples provided in the modules, and the outcome questionnaires. Through face-to-face consultations, participants from 2 stress management groups in regular cardiac rehabilitation, consisting of 8 MI patients each provided feedback on the overall design and content of the modules in focus group. The consultations led to following revisions: (1) simplified language, (2) revised examples of typical situations, reactions, thoughts, emotions, behaviors, and assignments, (3) inclusion of video clips of patients' stories on their experience of emotional distress after a myocardial infarction to the portal library, (4) improved technical functions related to login procedure such as instructions about how to retrieve of new password and how to get direct access to technical support, (5) different colors used in the platform, and (6) removal of a chat function. The chat function was removed since they described it as very unlikely that they would use such function. Feedback on the material was also obtained from two of the cardiac nurses involved in recruitment of patients at cardiac clinics. These face-to-face consultations led to revisions in the descriptions of common reactions and thoughts after a myocardial infarction, including more emphasis on fear and worry relate to having a new infarction.
