# Supplementary material for: Treatment Activity, User Satisfaction, and Experienced Usability of Internet-Based Cognitive Behavioral Therapy for Adults With Depression and Anxiety After a Myocardial Infarction: Mixed-Methods Study
Source: J Med Internet Res. 2018 Mar 16;20(3):e87. doi: 10.2196/jmir.9690 (PMC5878371; doi:10.2196/jmir.9690)
Supplement: Multimedia Appendix 3 [file jmir_v20i3e87_app3.pdf]

1. Tell me about how you feel right now.
2. When you first got into the program, what was your first impression?
3. What difficulties have you experienced using the portal?
4. Which parts of the program have you been spending time in?
5. What parts of the program did you find especially useful?
6. How much of the content of the program did you find to be relevant to your particular situation?
7. Was the program what you had expected?
8. What did you miss in the program?
9. How was the contact with your psychologist?
10. Do you have any suggestions for improvements to those who developed the program?
11. Is there anything else you would like to add?
