# Supplementary material for: Treatment Activity, User Satisfaction, and Experienced Usability of Internet-Based Cognitive Behavioral Therapy for Adults With Depression and Anxiety After a Myocardial Infarction: Mixed-Methods Study
Source: J Med Internet Res. 2018 Mar 16;20(3):e87. doi: 10.2196/jmir.9690 (PMC5878371; doi:10.2196/jmir.9690)
Supplement: Multimedia Appendix 4 [file jmir_v20i3e87_app4.pdf]

Meaning unit	Condensed meaning unit	Code	Sub-Category	Category
Logging in knowing I’d be asked loads of questions that would bring up lots of memories and feelings and thoughts, it was really hard to get going. I hesitated many times before logging in.	Logging in knowing I’d be asked questions that would bring up memories, feelings and thoughts, it was really hard. I hesitated many times before logging in.	Treatment rekindled difficult memories, thoughts and emotions.	Unpleasant emotions evoked by the intervention	Personal situation and required skills
In my case, I would need to learn about how to use a computer if I were to do this program. I am way too old to learn things like this. I just got an iPhone, if that’s what it’s called, from my daughter. She thought we could send pictures to each other and things like that. That is what I am talking from now. I get so upset, this gadget drives me crazy. It has its own mind. And my computer is the same.	I would need to learn about how to use a computer if I were to do this program. I am way too old to learn things like this. I just got an iPhone from my daughter. She thought we could send pictures to each other. I get so upset, this gadget drives me crazy. It has its own mind. And my computer is the same.	Low computer literacy	Technical issues	Personal situation and required skills
