# Supplementary material for: Treatment Activity, User Satisfaction, and Experienced Usability of Internet-Based Cognitive Behavioral Therapy for Adults With Depression and Anxiety After a Myocardial Infarction: Mixed-Methods Study
Source: J Med Internet Res. 2018 Mar 16;20(3):e87. doi: 10.2196/jmir.9690 (PMC5878371; doi:10.2196/jmir.9690)
Supplement: Multimedia Appendix 5 [file jmir_v20i3e87_app5.pdf]

The first author (EW), who was responsible for the primary analysis, is a licensed psychologist who worked as a therapist in the U-CARE Heart study. She identifies herself as female, and is in her mid 30s. She has previous experience of Cognitive Behavior Therapy delivered face-to-face for common psychological problems, including symptoms of depression and anxiety. She has previous experience in conducting inductive content analysis.

The fourth author (GB), who conducted the telephone interviews, is a researcher and a licensed psychologist. She identifies herself as female, and is in her mid 60s. She has previous experience in stress management groups among cardiac patients. The interviewer was not involved as a therapist in U-CARE Heart trial.

The last author (TC), who was responsible for providing feedback on the primary analysis, is a specialist intensive care nurse and registered midwife. He is a researcher with formal training and experience with qualitative content analysis. He identifies himself as male and is in his 30’s. He has previous clinical experience in intensive care of patients with acute myocardial infarction and heart failure, and has no clinical experience of rehabilitative cardiac care following a myocardial infarction. He has experience as a teacher in cardiac care for bachelor’s level nursing students. Before the analysis, he had no experience with the U-CARE Heart intervention.

All authors are born in Sweden.
