# Supplementary material for: Treatment Activity, User Satisfaction, and Experienced Usability of Internet-Based Cognitive Behavioral Therapy for Adults With Depression and Anxiety After a Myocardial Infarction: Mixed-Methods Study
Source: J Med Internet Res. 2018 Mar 16;20(3):e87. doi: 10.2196/jmir.9690 (PMC5878371; doi:10.2196/jmir.9690)
Supplement: Multimedia Appendix 6 [file jmir_v20i3e87_app6.pdf]

Treatment week	Completed modules			Completed assignments			Messages sent to therapist		
	n	M(SD)	Range	n	M(SD)	Range	n	M(SD)	Range
1	5	0.04(0.20)	0-1	65	0.56(0.90)	0-5	14	0.12(0.44)	0-3
2	6	0.05(0.22)	0-1	36	0.31(0.64)	0-3	17	0.15(0.44)	0-2
3	7	0.06(0.24)	0-1	33	0.28(0.67)	0-3	15	0.13(0.45)	0-3
4	7	0.06(0.24)	0-1	23	0.20(0.50)	0-3	12	0.10(0.42)	0-3
5	5	0.04(0.20)	0-1	26	0.22(0.63)	0-4	14	0.12(0.40)	0-3
6	8	0.07(0.31)	0-2	38	0.32(0.69)	0-3	17	0.15(0.44)	0-2
7	12	0.10(0.36)	0-2	27	0.23(0.59)	0-3	8	0.07(0.29)	0-2
8	6	0.05(0.29)	0-2	14	0.12(0.44)	0-3	8	0.07(0.25)	0-1
9	7	0.06(0.30)	0-2	20	0.17(0.58)	0-4	7	0.06(0.27)	0-2
10	7	0.06(0.33)	0-2	9	0.08(0.30)	0-2	10	0.09(0.31)	0-2
11	3	0.03(0.21)	0-2	17	0.15(0.71)	0-7	12	0.10(0.40)	0-2
12	5	0.04(0.24)	0-2	13	0.11(0.43)	0-3	2	0.02(0.13)	0-1
13	7	0.06(0.33)	0-2	17	0.15(0.44)	0-3	9	0.08(0.30)	0-2
14	4	0.03(0.22)	0-2	23	0.20(0.94)	0-7	21	0.18(0.78)	0-7
Total	89	0.76(0.93)	0-5	361	3.09(4.05)	0-29	166	1.42(2.56)	0-16
