# Supplementary material for: Treatment Activity, User Satisfaction, and Experienced Usability of Internet-Based Cognitive Behavioral Therapy for Adults With Depression and Anxiety After a Myocardial Infarction: Mixed-Methods Study
Source: J Med Internet Res. 2018 Mar 16;20(3):e87. doi: 10.2196/jmir.9690 (PMC5878371; doi:10.2196/jmir.9690)
Supplement: Multimedia Appendix 7 [file jmir_v20i3e87_app7.pdf]

Category	Sub-category	Experiences	Findings	n	Illustrative quote
**Portal**					
	Design	Positive	Easy to navigate the different sections	4	*It was easy to navigate.* [3560]
			Appealing interface	2	*The design was very well done.* [4044]
		Negative	Navigational difficulties, especially in beginning of treatment	8	*It took me a while to figure out how the program was organized and that made it hard at the beginning.* [4148]
			Unfamiliar interface	1	*I did not feel at home with it.* [3132]
	Usability	Positive	Easy login procedure	2	*It wasn't hard to find the program, log in, or figure out how it worked.* [4098]
			Secure access via two-factor authentication solution	1	*The high level of security while logging in made me feel safe.* [4553]
		Negative	Complicated login procedure	3	*The login procedure felt a little too modern for us old folks.* [3934]
			Technical failures related to the login procedure	3	*Had a lot of technical issues from the start. Had trouble logging in.* [4148]
			Required access to desktop or laptop computers	2	*Since I worked on this program on my home computer, I often did it late in the evening. If it had been adapted to my mobile phone I think I would have worked with it more often.* [3560]
			Technical issues when accessing on mobile devices	2	*Didn't do so much since I can't write on my iPad.* [4471]
			Cumbersome to open the PDF-files	1	*The system with all the attachments was*

			used in the treatment		*cumbersome, all the PDF-files that you had to open.* [3132]
**Treatment program**					
	Content of the treatment material	Positive	Relevant and useful information	19	*I felt that it [the content] was spot on for me.* [4168]
			Well-written material	3	*I was surprised that the material in the program was so nicely presented.* [4553]
		Negative	Irrelevant modules, examples, and overall focus	9	*It felt like there was an underlying assumption that anxiety was caused by the heart attack. This is not the case for me.* [3132]
			Outdated and irrelevant posts in the discussion board	4	*Couldn't really relate to the Forum part. Most of what was posted there was old.* [3560]
			Poor overall readability	2	*The way the text was written made it very hard to understand.* [4471]
			Repetitive material	2	*There was a lot of repetition between passages.* [4168]
	Working with the material	Positive	Overall manageable degree of difficulty	2	*There was an adequate amount of text and good level of difficulty. Not too advanced but not too shallow either.* [4249]
			Text-based approach gave time to reflect about their own behavior	6	*Reading the text and exercises helped me to write down a description of my own problem. […] I got a better picture of it.* [4532]
			Time flexibility and working at their own pace	3	*I liked that you didn't have to finish an exercise all at once but you could push the 'continue later' button.* [4342]
			Possibility to select relevant modules and tailor the content according to own needs	6	*Being able to ask my own questions, figure out my own answers and then create my own treatment was very useful.* [4532]
		Negative	Strenuous and time-consuming work	11	*You could register over several weeks. I managed to do that 4 times. I just felt, this isn't for me.* [4553]

			Tedious and boring work	4	*It was a bit boring.* [4093]
			Working on you own was demanding	3	*Getting started with these assignments demanded a lot of high level insight and self-discipline.* [3129]
			Difficult to write about thoughts and feelings	8	*I don't feel like I could really express what I wanted to say. I am more a verbal type of person.* [4148]
			Difficult to find a balance between working on your own and therapist support	2	*There was something in this balance between a self-help manual on the computer and an active dialogue with a psychologist that made it hard for me.* [3129]
			Too intensive to work with one step per week	5	*Did not expect the pace to be so fast.* [4553]
			Restrictions in number of simultaneous active modules felt rigid	3	*I didn't like that I had to finish each module before I could go on to the next. I would have liked to have an overview.* [4148]
	Treatment period	Positive	Having a deadline promoted activity towards end of treatment	2	*I got a lot more active when the time ran out.* [4249]
		Negative	Duration of treatment and time to work with modules too short	4	*There was a shortage of time. Maybe it would have felt less intense given more time.* [3132]
**Therapist communication**					
	Therapist feedback	Positive	Individualized feedback	12	*Still, the responses I got felt like I had been understood. The responses felt relevant to me personally.* [4249]
			Telephone conversations with therapist	7	*She called me a few times and I got to tell my whole story. That felt good.* [3132]
			Available feedback	6	*I felt that I got active support when I got working on the assignments.* [3129]
			Reminders useful prompts to login	3	*It took me a long time to log in until I finally got a reminder on text message. This made me feel guilty and I logged in. Good with those reminders.* [4532]

			Rapid feedback	2	*It took less than 24 hours to get feedback.* [4532]
		Negative	Lack of therapist feedback	9	*These types of exercises or programs demands more motivation or support.* [3560]
			Aversive and stressful reminders	4	*They tried to call me a few times because I had responded to the modules. This made me feel guilty and it made everything even harder.* [3132]
			Irrelevant feedback	3	*I asked which [module] do you think I should choose. Got a suggestion to work with module I absolutely did not want to work with so I skipped that module and then I had a hard time to start up again.* [3729]
	Web-based communication	Negative	Preference for verbal and synchronous communication	9	*When I write it takes a lot of time and it feels awkward, it is always easier to talk to somebody than to write.* [3774]
			The text-based communication felt impersonal	2	*In this case I had a formed a relation with her [former therapist outside U-CARE] and you don´t get that in a flat text.* [4249]
			Possible risk of misunderstandings	1	*[To] write can be misunderstood, when you talk you can hear the nuances in how words are expressed.* [3774]
**Personal situation and required skills**					
	Unpleasant emotions evoked by the intervention	Negative	Bad conscience and guilt for being inactive	6	*Feeling guilty was my motivation for logging into the program. I only did the bare minimum to get by.* [3120]
			Treatment rekindled difficult memories, emotions, and thoughts	4	*It evoked a lot of memories, emotions and thoughts. It was really hard in the beginning.* [4532]
			Fear of making mistakes	1	*You were afraid to make a fool of yourself and to make mistakes.* [3739]
	Lack of time	Negative	Lack of time because everyday	7	*There were loads of assignments that were very time*

		chores, children, and working fulltime		*consuming. I felt like I just didn't have that time since I work full time and have kids at home.* [4553]
		Poor timing of treatment	2	*I had [already] gathered information on my own […] so by now I already have some insight.* [3934]
Responding to outcome measures in intervention study	Negative	Self-reports were difficult to understand	1	*Sometimes I could not figure it out. What do they want, what do they want me to write […] I would have preferred it more simple, now it was very academic.* [3739]
		Self-reports felt repetitive	1	*Many of the questions were repetitive, it was the same questions over and over again.* [4044]
		Strenuous work to respond to self-report measures	2	*There were many forms to fill out, too many maybe.* [3132]
		Self-report measures felt irrelevant	5	*There were many questions that were not relevant for me, maybe relevant for older people, can you dress yourself, take care of your personal hygiene, can you take your medicine.* [3132]
Technical issues	Negative	Low computer literacy	2	*How is it possible, for me who is not computer savvy to get a grip on this?* [3739]
		Intervention requires them to spend time in front of a computer	2	*It took a lot of time and I spend all day in front of a computer. I really didn't want to sit with the computer in my spare time.* [3132]
		Lack of Internet connection	2	*I spent a lot of time last summer at in my summer cottage where there is no internet connection.* [3120]
